# The Hahnemann Medical College

**Published:** 1868-05

**Authors:** 


*The Hahnemann Medical College* building in Chicago, has been seized by the officers of the United States government, it having been used as a cover to a *vinegar factory*, in which the shrewd detectives found a concealed still, with all its appurtenances. It is claimed, however, that the still was onty in use for preparing "mother tinctures" and attenuations. There is much consternation in homœopathic circles in the city over this *mesalliance* of vinegar, whisky, law and infinitesimals. The Faculty refuse to testify as *experts* in the litigation which is to follow unless allowed honorary fees.—*Chicago Medical Journal.*

